# Development of Clinically Relevant Implantable Pressure Sensors: Perspectives and Challenges

**DOI:** 10.3390/s140917686

**Published:** 2014-09-22

**Authors:** Ingelin Clausen, Thomas Glott

**Affiliations:** 1 SINTEF ICT, Department of Microsystems and Nanotechnology, NO-0314 Oslo, Norway; 2 Sunnaas Rehabilitation Hospital HF, NO-1450 Nesoddtangen, Norway; E-Mail: thomas.glott@sunnaas.no

**Keywords:** implantable MEMS, pressure, sensor design, protective coatings, clinical relevance, clinical study

## Abstract

This review describes different aspects to consider when developing implantable pressure sensor systems. *Measurement of pressure* is in general highly important in clinical practice and medical research. Due to the small size, light weight and low energy consumption Micro Electro Mechanical Systems (MEMS) technology represents new possibilities for monitoring of physiological parameters *inside* the human body. Development of clinical relevant sensors requires close collaboration between technological experts and medical clinicians. Site of operation, size restrictions, patient safety, and required measurement range and resolution, are only some conditions that must be taken into account. An implantable device has to operate under very hostile conditions. Long-term *in vivo* pressure measurements are particularly demanding because the pressure sensitive part of the sensor must be in direct or indirect physical contact with the medium for which we want to detect the pressure. New sensor packaging concepts are demanded and must be developed through combined effort between scientists in MEMS technology, material science, and biology. Before launching a new medical device on the market, clinical studies must be performed. Regulatory documents and international standards set the premises for how such studies shall be conducted and reported.

## 1. Introduction

Due to the small size, light weight and low energy consumption Micro Electro Mechanical Systems (MEMS) technology represents new possibilities for monitoring of physiological parameters *inside* the human body. Combined with constant advances in technology for wireless energy and data transmission, long-term *in vivo* measurements are achievable. Such measurements are expected to improve the quality of medical diagnosis and treatment. *Measurement of pressure* is in general highly important in clinical practice and medical research. Pressure in the circulatory system, intraocular, urinary bladder, muscle compartments, joints (e.g., knee and hip) and brain are only some examples of pressures being routinely measured. Although such *in vivo* pressure measurements are regularly carried out, they are currently limited to a short period of time (e.g., during or after surgery) due to patient comfort and safety. In addition, common measurements in clinical practice do not reveal the exact pressure at the site of interest, but represents only the best achievable measure of the patient condition.

In this review we focus on the importance of long-term *in vivo* pressure measurements in clinical practice, and how novel technology in this field can promise immense healthcare improvements. The potential for implantable pressure sensor technology is huge. The technology is presently at an early development stage. The ultimate implantable sensor system is wireless, with the sensing and the electronic parts placed inside the human body, and can stay in place and work properly for years. Or even more advanced: an actuator is included so the device can measure and perform an active operation, e.g., electrical nerve/muscular stimulation. On the route to this ultimate solution, wired miniaturized sensors providing high-quality measurements for shorter periods of time might still offer sufficient functionality. PC-connected solutions might be suitable for bedridden patients for measurements up to 48 h. Portable logger might be suitable for monitoring of inpatients for some weeks.

The traditional division between medicine and technology is a major challenge in development of new devices. Experts are rarely collocated. Medical clinicians lack the knowledge of potential in novel technological solutions. Technological experts are not aware of which medical developments it is possible to achieve. This may result in technical gadgets with no clinical relevance.

This review is based on selected papers in technological and medical literature and on experience gained during the development of MEMS based *in vivo* technology for monitoring of pressures in the brain, big joints and in the urinary bladder. Some specific challenges in the development of implantable pressure sensor systems are described along with current status in this field. Design issues, biocompatibility, packaging, safety and regulatory affairs are covered, while powering and signal readout is only briefly mentioned. Although pressure measurements are extensively carried out in clinical practice, there has not been a strong tradition for exchange of experience between each field of medical disciplines nor between technologists and physicians. We hope that this review can provide for exchange of experience both between and across the different fields of expertise.

## 2. Terminology and Definitions

Development of implantable pressure sensor systems is a highly multidisciplinary task. After working in this field for more than a decade we are aware that some common terms have a somewhat different meaning within the separate fields of expertise. In this paper we consistently use the term *sensor element* for the transducer or silicon chip, *sensor* is used for the transducer including electronics (signal conditioning unit and AD converter), and *sensor system* is used for the complete system including sensor and communication or readout unit, where the communication unit delivers energy to and transfers data from the sensor.

Also some attention must be paid to the definition of *implantable devices*. The term seems not to be totally clear. ISO 13485 [1] defines an active implantable medical device as a medical device that uses electricity or other energy, is partly or totally inserted into the human body or a natural orifice by means of surgical or medical procedures, and is expected to stay there after the procedure is completed.

In the FDA list of investigational device exemption (IDE) an implant is defined as a device that is placed into a surgically or naturally formed cavity of the human body and is intended to remain there for a period of 30 days or more [2]. However, in order to protect public health, FDA may determine that devices placed in subjects for shorter periods are also implants.

ISO 10993-1 describes categorization of medical devices based on the nature and duration of their contact with the body [3]. Categorization by the nature of body contact separates the devices into non-contacting devices, surface contacting devices, external communicating devices, and implant devices. Devices contacting intact mucosal membranes, e.g., urinary catheters, are classified as surface contacting devices and not as implant devices. Categorization by the duration of contact divides the devices into category A—Limited exposure: devices whose single or multiple use or contact is likely to be up to 24 h; category B—Prolonged exposure: devices whose single, multiple or long-term use or contact is likely to exceed 24 h but not 30 days; and category C—Permanent contact: devices whose single, multiple or long-term use or contact exceeds 30 days.

In this paper we follow the ISO 13485 definition for an active implantable medical device. However, we use the term subcutaneously implantable for medical devices being totally inserted into the human body and the term percutaneously implantable for devices with wires penetrating the tissue/skin for connection to an external energy source or readout unit. We also follow the more precise definition in ISO 10993-1 for the duration of contact with the body: permanent use exceeds 30 days. Thus, with the term subcutaneously implantable device we mean a medical device being totally inserted into the human body for more than 30 days. We use the term *in vivo* for any device used in the human body, irrespective of being defined as implantable.

## 3. Why Are Pressure Measurements Important in Medical Practice?

Pressure is one of the vital parameters for any living organism. Evolution has devised countless ways of preserving homeostasis within an organism's habitat. In the human body, pressure is an essential parameter in almost all organs. Deviation of pressure out of range for physiological function may result in injury or deteriorating function. In the following we highlight some relevant aspects of pressure in the human body, and its importance to physiological functions.

Pressure in the human body is influenced both by external and internal factors. Examples of external factors are forces due to atmospheric pressure and gravity. Atmospheric pressure does not represent any challenge except in situations where normal limits are exceeded, like in diving or space flight. The life-threatening situation occurring in decompression sickness is well known, and measures to counteract these effects have been known for centuries. Gravity is a ubiquitous force continually exerting pressure on our body during our lifetime. This tallies with the fact that degenerative changes are most prevalent in weight bearing joints. When falling, exceeding physiological limits may cause soft tissue injury and fractures. Measurement of forces and pressure acting during an injury are extremely difficult, and is usually done by calculating change in velocity. However, there is a considerable uncertainty about the forces and pressure acting inside the body part itself.

Examples of internal factors are forces generated by the action of muscle. This may be volitional like in striated muscles, or autonomous like cardiac, vascular, intestinal or bladder muscle. Pressure exerted by uncontrolled muscular activity, such as spasticity, may cause severe deformity. The function or dysfunction of the cardiac muscle and blood vessels are connected to a number of common and severe health problems like hypertension, cardiac failure or infarction. Intestinal muscle dysfunction may cause problems like incontinence or constipation, while bladder dysfunction may affect the kidney function. The autonomous nerve system has pressure sensors for blood pressure, intended for reflex homeostasis. However, even in severe cases there are usually few or no symptoms for the person affected.

Pressure is also an important part of our sensory system. There are several different mechanoreceptors in the skin responding to external pressure or vibration. This sensation is vital for preservation of skin integrity. In patients with neurological conditions, lack of pressure sensation is a common cause of chronic skin ulcerations.

On a cellular level, pressure is important for osmosis of fluids, oxygen and nutrients. These pressure gradients are usually much lower than the previously mentioned. Perfusion of blood into small vessel carrying oxygen and nutrients may be obstructed when the organ's pressure exceed perfusion pressure. The brain is encaged in a skull, and any space occupying process like hematoma or swelling of the brain tissue, will increase the internal pressure. When the internal pressure in the cerebrospinal fluid exceeds the blood's perfusion pressure, supply of vital oxygen will be obstructed. In worst case, blood flow is completely obstructed, which in 10–15 min results in a “*brain death*”. The same scenario applies for part of the striated muscles being in compartment of fascia. Although striated muscle is more robust, loss of blood supply may eventually lead to damage of the muscle with pain, paralysis and inflammation.

## 4. Technological Development

Blood pressure was first described already in the 17th century. Some improvements have been introduced since then; however, clinical measurement techniques have been based on the same principles for decades. Being the most common pressure measurement in clinical practice, other procedures often have been based on the same technology. There are several challenges in recording pressure in general: examinations represent a snapshot covering only a few seconds. The measuring device may in itself cause artifacts. These may be psychological, like the well-known “*white collar hypertension*” or physiological through interfering with reflex functions through sensory mechanisms.

While pressure measurement has been part of routine practice in many different disciplines of medicine, it has been extremely difficult to determine exact ranges with regards to risk. In most cases, there are wide ranges, divided in several steps like normal, low-risk and high-risk. Efficacy of treatment has been difficult to establish, both in studies as well as in the individual patient. This situation may be improved by better pressure measurement technology.

In current clinical practice pressures are often measured indirectly through a water or air column and/or at a location remote from the site of interest. Thus, optimal real-time measurements are not provided and the exact pressure inside the organ of interest is not revealed. Also, patient discomfort, risk of infections, and reflex activity to the smooth muscle are associated with existing methods, e.g., when measuring pressure through the urethra. Pressure measurement is also prone to artifacts from movement, and usually has to be done in a standardized situation like sitting still on a chair. As a consequence, such methods are not suitable for long-term recordings during normal activities.

*In vivo* pressure sensors are routinely used during surgery, for a short period after surgery, or for immediate inspection, for different clinical applications like brain pressure monitoring and diagnosis of urinary bladder complications. Some of these sensors are referred to as microsensors [4,5], but being connected to external power and/or readout units through wires or cables they are not suitable for permanent implantation. Besides, problems with existing technology in clinical practice are reported. Eide and co-authors have described in several papers sudden shifts or gradual drifts in baseline pressure when monitoring intracranial pressure [6–8]. Some of the reported problems are of technical origin, e.g., electrostatic discharge. The alterations in baseline pressure are clinically relevant and would affect patient management. Therefore, not only improved technology offering the possibility for totally new clinical procedures and for continuously monitoring organ functions are strongly desired by the medical community, but also technology providing for more reliable measurements in today's clinical practice.

Research on permanently implantable blood pressure sensor for monitoring of hypertension has been in progress for many years [9–12]. The situation is similar for the development of an implantable intraocular pressure sensor for detection of glaucoma [13–17]. Implantable pressure sensors for measurement of intracranial pressure have also been subject of research and development for decades [18–22].

Although implantable pressure sensors have been the topic of intensive research and development for many years, only a handful of devices have been the subject of clinical studies. A nice review of implantable sensors for monitoring of heart failure is given by Merchant and co-authors [23]. Some implantable sensor systems for cardiovascular applications are in various stages of clinical testing [24–28]. Quite recently (June 2014), implantation of a wireless intraocular pressure transducer in the human eye was reported [29]. A clinical study is now carried out at glaucoma patients [30]. However, until earlier this year, the only commercially available implantable pressure sensor with approval for permanent implantation was the EndoSure® Wireless AAA pressure sensor from CardioMEMS (Atlanta, GA, USA). This device was left behind in the aneurism sac for possible post-surgical pressure measurements. The authors of this paper do not know the current status of this technology.

These days there seems to be a breakthrough for the first permanently implantable pressure sensors to be launched on the market. In May this year the CardioMEMS™ HF System for heart failure management (a slightly different version of the above described CardioMEMS technology), received FDA approval. A present, FDA is requiring a thorough post-approval study to gain more information about the device's performance when used outside the context of a clinical study [31].

## 5. Physical Principles of *in vivo* Pressure Sensing

Generally there are three main measurement principles applied for *in vivo* pressure sensors:

*Fiber optic* sensors quantify the modulation of light through the fiber caused by external pressure, or convey the light from a remote sensor to the electronics that process the signals. Fiber optic sensors were introduced several decades ago. Such sensors benefit from small size and high elasticity, are immune to electromagnetic interference, and are demonstrated to be MR-compatible [32]. Fiber optic catheter—tip pressure sensors are used for intravascular blood pressure monitoring, muscle compartment pressure monitoring, intracranial pressure monitoring, and for intraocular pressure measurements [33]. Fiber-optic sensors can be made small, for example is the FOP-F125 from FISO (Québec, QC, Canada) claimed to be the world's smallest pressure sensor, having a diameter of only 125 μm. The disadvantage with fiber optic sensors is the fiber through the skin, representing a risk of infection and meaning that the system cannot be made subcutaneously implantable (*ref.* Section 2).

*Capacitive* pressure sensors determine the diaphragm displacement, caused by a pressure, as a change in capacitance. This measurement principle is known to be especially effective for the measurement of low pressures [34,35], but is also known to be vulnerable to parasitic capacitances in electrical wires [36]. Thus, necessary electronics must be located close to the sensing part [36,37]. Even if the sensor element itself (*i.e.*, the transducer) can be made small, the resulting sensor size (sensor element + electronic chip) will be too large for many *in vivo* applications (*ref.* Section 6). The measurement principle of the CardioMEMS™ HF System, however, is capacitive. Energy delivered to and data collected from the implanted device is done by telemetry. Although the sensor chip is relatively large, 3.5 × 15 × 2 mm [38], it is still small enough to be inserted into the pulmonary artery. The measurement principle of the implantable intraocular pressure sensor device is also capacitive [29]. Energy to and data from the wirelessly implanted device is delivered by inductive coupling. The size of this sensor chip is also relatively large with an outer diameter of 11.3 mm. However, the sensor size is small enough to be placed in the sulcus space of the human eye and therefore adequate for the specific application. *Piezoresistive* pressure sensors detect the bending of a diaphragm as a change in resistance in the piezoresistors embedded in the diaphragm. Although more vulnerable to electric noise than capacitive pressure sensors, piezoresistive pressure sensors have their advantage in *in vivo* applications because any necessary electronics may be separated from the pressure sensor element and placed at a location where the space restriction is less severe [39]. Due to the smaller size of the separate sensor element it might fit well into a catheter or a tube inserted into a body cavity. A typical size of the inner diameter of a ventricular catheter for intracranial pressure management is 1.5 mm. A piezoresistive solution also provides for easier wireless transfer of energy to, and data from, the sensor element, because the electronics can be placed directly under the skin. An inductive method for energy and data transmission might therefore be applied also for applications where the sensor element is implanted deeper into the body than, e.g., the eye sulcus space.

## 6. The Ideal Pressure Measurement Scenario from a Clinical Point of View and Concerns for Sensor Development

From a clinical standpoint, the ideal pressure measurement is done for a longer period of time with as little discomfort as possible for the patient. Assessing physiological processes usually requires several days of recording, during different activities normally performed by the person. Measuring efficacy of treatment in an individual patient may require weeks of measurement, while preventing measures may be for months or even years.

Avoiding discomfort may be achieved by placing the pressure sensor as inert as possible with no risk for displacement or interference with pain receptors. The smaller the device, the less is the risk for tissue damage causing inflammation and pain. Reducing inflammation may also prove important to prevent fibrosis and calcification interfering with the sensor. In general, a miniature device placed inertly and deep may prove most acceptable.

Not surprisingly, therefore, is the physical size the overriding design issue for an implantable pressure sensor. Both to achieve minimally invasive procedures and to obtain monitoring possibilities in vulnerable body organs the size must be small. What is meant exactly by small depends on the specific application, as illustrated by the examples given in the previous section.

Pressure range and measurement resolution (thus sensor sensitivity) are other important qualities. The measurement must be precise in the relevant interval for the organ being studied. We suggest dividing the pressures in the body into three domains: *low pressure domain* (capillaries, brain, urinary bladder, and muscular compartments); *medium pressure domain* (circulatory system including the heart), and *high pressure domain* (load bearing structures like hips and knees). Historically a great variety of units have been used for expressing pressure, depending on their suitability for the application. In Bosch Kraftfahrtechnisches Taschenbuch from 1961 [40], a conversion table shows no less than 13 different units of pressure, the SI unit pascal (Pa) not being included as this was first introduced in 1971. A more updated conversion table containing eight different pressure units can be found in Fraden's Handbook of Modern Sensors [34]. Although being the accepted scientific unit Pa is hardly used among medical experts. Pressure exerted from a column of water was from the beginning the preferred method in clinical practice, and was used for low pressures like brain pressure. For higher pressures, like blood pressure, the height of the water column would simply be too high and mercury manometers were then introduced. Brain pressure is still measured in mmH_2_O while mmHg is the common unit for expressing blood pressure. Not less confusing; Sensor manufacturers commonly use the unit bar. A compromise for communication among medical experts and sensor developers might be mbar, also ensuring a link to the scientific unit through the relationship 1 mbar = 100 Pa.

Typical values in the low pressure domain are between 0 and 10 mbar (1 mbar = 100 Pa ≈ 10 mmH_2_O), in the medium pressure domain the values lie between 25 and 250 mbar (1 mbar = 100 Pa ≈ 0.75 mmHg)), while in the high pressure domain the values can be as high as 180 bar (18 MPa) [41]. The required measurement resolution might vary depending on the application, but a typical value is 1 mbar. High quality measurements may require reference pressure recordings in addition to the target pressure (*i.e.*, the pressure in the organ subject of investigation) with adequate sampling rate and precise timing. The ideal location of reference measurements might be in the tissue close to the organ, but for e.g. intracranial pressure monitoring the relevant reference pressure is the atmospheric pressure.

Other considerations when designing an implantable sensor system are currents and voltages delivered to the patient, heat generated in the human body, and possible mechanically introduced harm and injuries caused on tissue/skin. Both normal operation and possible sensor system malfunction must be evaluated. Risk management must be employed during the complete system development run [42]. Standard current limits must be respected [43].

## 7. The Specific Challenges with *in vivo* Pressure Measurements

Any implantable device has to operate under very hostile conditions inside the human body; a humid environment at 37 °C and with proteins, enzymes, cells, inorganic and organic ions. In the sensor community it is well known that *in vivo* pressure measurements are particularly demanding because the pressure sensitive part of the sensor must be in direct or indirect physical contact with the medium for which we want to detect the pressure. The options for protecting an implantable pressure sensor towards body attach are therefore limited. This is in contrast to measurement of, e.g., acceleration, where direct contact with the surrounding fluid or tissue is not required.

Any foreign implantation may evoke immunologic responses. The reaction may depend on the properties of the implant (size, structure, material), but also on the individual's immune system. Local inflammatory responses activate the immune system with cells like macrophages and leukocytes. In addition, a wide range of inflammatory mediators are released with various local effects. Additionally, a foreign body may cause local bleeding. Although minute in volume, the blood clot may also cause local inflammation and fibrosis. Moreover, any implant has the risk of introducing and being colonized with bacteria. A bacterial infection may be due to inadequate sterilization, but may also be blood borne (hematogenous transfer). Besides causing a local swelling, pain and redness, any infection will also stimulate the immune system additionally, again causing formation of fibrous tissue. Thus, in general any foreign implant is at risk of being encapsulated in fibrous, or “scar tissue”.

Immunologic responses are regarded a major obstacle for the success of implantable sensors in the human body [44]. One condition of particular concern regarding pressure sensors for long-term implantation is biofouling, *i.e.*, the adhesion of proteins and other biological matter on the pressure sensitive part of the sensor element. The immunologic processes might be dynamic, resulting in varying thickness of the layer(s) being accumulated on the surface and thereby varying the sensor output signal [39]. Thus, for the development of a permanent implantable pressure sensor drift caused by biological processes is a main concern. Correspondingly, final fibrous encapsulation might result in sensor failure. Also corrosion caused by aggressive body fluids may alter the sensor characteristics and thereby sensor stability and again result in sensor failure. The challenges related to immunologic responses might be overcome by smart packaging with novel biocompatible surface coatings that eliminate, e.g., protein adsorption. This is discussed in more detail in Section 8, Packaging.

To a certain degree the site of operation determines the challenges imposed by the body environment. The environment the sensor will ‘see’ when implanted depends very much on the localization within the body. The body temperature is mostly stable at 37 °C, but other parameters like pH and presence of cells and proteins will vary depending on implantation site. In general, the most demanding site of operation is the cardiovascular system. If the sensor is in direct contact with blood it will be exposed to blood platelets, cells and proteins that will react with the sensor surface. The salt content in the blood is 0.9% and might give rise to corrosion. An overview of the different types of environments within the body is given in Table 1.

Some of the immunologic responses may be reduced by a smart sensor design. Our own group has developed a miniaturized piezoresistive sensor element which is unique with respect to protection of the piezoresistors without diminishing the sensitivity; the piezoresistors are placed at the diaphragm surface facing the vacuum reference cavity closed by the anodic bonded glass wafer [39]. The piezoresistors are therefore not in contact with the biological environment. Furthermore, a protective coating can be added to the diaphragm without moving the piezoresistors closer to the neutral plane of the diaphragm [45]. A decrease in sensitivity due to a protective coating is therefore reduced.

Schurr and colleagues have described an implantable telemetric blood pressure sensor placed on the outer surface of an artery [46]. The measurement approach is to measure the wall tension, which correlates with the intravascular pressure. Thus, direct contact with the inner surface of the arteries is avoided.

Although some of the challenges can be overcome by smart sensor design, successful development of novel medical technology depends on collaboration between experts in technology and medicine. Technological experts may not be aware of which medical developments it is possible to achieve. Medical technology development by technological experts alone may therefore result in technical gadgets with no clinical relevance.

## 8. Packaging

Packaging of MEMS devices is in general a critical step for the final application. For medical *in vivo* sensors the situation is particularly demanding. The package has to provide for electrical connection, it has to provide an adequate path for heat generated by power dissipation, and it must protect the sensor element. At present the only commercially available long-term *in vivo* sensors are those that are protected by (bulky) metal casings, like the accelerometers of pacemakers and defibrillators. The reason for this is most probably due to their sensing nature which allows for more direct means than, e.g., for pressure sensors.

Strict requirements are forced upon the materials to be used for packaging of an implantable pressure sensor. A proper packaging solution must offer: (i) biocompatibility such as anti-inflammatory qualities and body-mimicking properties, and (ii) biostability by providing antifouling properties and long-term functionality and stability of the implantable device [47]. The packaging material must provide for minimum expected lifetime and have sufficient protection properties. Equally, important qualities from the sensor point of view; the packaging solution must not cause any critical degradation of sensor performance and characteristics. Of them size, sensitivity, and stability are the most important ones.

The relatively massive cages or housings of metal or solid polymers traditionally used for MEMS packaging will in many cases spoil the opportunities of miniaturized implantable medical MEMS. Protection of the sensor element surface with a thin biocompatible coating might provide sufficient protection against biological attack, and at the same time maintain the small-size advantage. Our group has for several years examined biocompatible coatings of nanoscale thickness as a substitute for the traditional MEMS packages. Changes in device characteristics after depositing thin biocompatible coatings by Atomic Layer Deposition (ALD) were investigated [48]. Also device characteristics were examined after submersion in human liver extract [49] and after more than 30 days submersion in true human synovial fluid [50]. The results were promising for the development of miniaturized sensors for long-term *in vivo* measurements, although realistic *in vivo* tests are still required.

The success of *in vivo* sensors partly depends on the availability of biomaterials that are biocompatible, body mimicking, and stable in the long term. There has for a long time been intensive research on finding fouling-resistant and specifically protein-resistant surfaces [47]. Such surfaces are desired in a large variety of industrially or medically important situations. Of several chemical groups polyethylene glycol (PEG) has proven to have the most protein-resistant functionality and remains the standard for comparison [51]. Research is in progress to minimize protein adsorption and cell adhesion in microfluidic devices, where clogging of the microfluidic channels as well as unwanted removal of the analytes from solutions are recognized challenges [52–54]. Extensive research has also been carried out for implantable devices as stents and heart valve prosthesis [55,56].

The biomaterial coatings must be designed to fit underlying materials, specific geometries and systems of different complexities. The thin film coatings should be evaluated with a whole range of biological assays providing information on the processes taking place at the interface between coating materials and the tissue. In situ methods might be an efficient tool to reveal information on mass and structure of formed layers (e.g., proteins and lipid bilayers) and the ingrowth process [57–59]. To succeed with the development of new biomaterials suitable as coating for implantable sensor, extensive cross-disciplinary collaboration among MEMS developers, experts in material science and biologists are required.

## 9. Testing

A sensor designed for *in vivo* applications must go through a range of tests to be approved as a medical device. The type of tests will differ for different applications, depending on implantation site, physical layout of sensor, functionality etc. The required test program must be developed for the specific device. Some of the mechanisms leading to sensor failure might be accelerated in *in vitro* experiments. Corrosion or mechanical wear out are examples of such mechanisms. The major challenge is to make the experiments (especially in vitro) representative and realistic. We must differentiate between testing the biocompatibility of a device and testing the functionality and reliability of a device. Biocompatibility testing should be performed according to the ISO 10993-1 [3]. Even if we use materials already approved for *in vivo* use it might be necessary to perform biocompatibility testing.

### 9.1. In Vitro versus In Vivo Testing

Yang *et al.* have discussed *in vivo versus in vitro* testing [60]. According to Yang *in vitro* tests may serve as precursors for more involved, more costly and time consuming animal trials. One should however be aware that *in vitro* experiments may give different results than *in vivo* since it is not possible to reproduce the complete environment around the device in an *in vitro* test. *In vitro* tissue culture experiments are considered by many to be too sensitive when used alone, and many materials that are used *in vivo* today would be dismissed by this type of *in vitro* testing. The living cells in the body have the capability to buffer local effects around the implant site, while this will not occur *in vitro*.

Ratner [47] gives one example of different *in vivo* and *in vitro* material response: tissue culture polystyrene, a surface modified polymer, will readily attach and grow most cells in culture. Untreated polystyrene will neither attach nor grow cells in vitro. However, when these two materials are implanted, both materials get covered by a thin foreign body capsule and are almost indistinguishable.

*In vitro* testing simulating thrombogenicity in a blood flow (*i.e.*, risk for blood clotting) is especially challenging since the result depends on many factors like flow rate and blood chemistry. There is also a risk of getting non-conclusive results or even wrong conclusions due to misinterpretation of results [47].

*In vitro* tests are nevertheless useful in early evaluation and screening of materials and devices. They minimize the use of animals in research and are much less expensive than *in vivo* animal experiments.

### 9.2. Clinical Studies

The safety, performance and efficacy of a medical device are mandatory to know for patients and health personnel. Before launching a new medical device on the market, clinical studies must therefore be performed. Regulatory documents and international standards set the premises for how such studies shall be conducted and reported [42,61]. However, there are several challenges in designing and completing a clinical study on an implantable device. The number of clinical studies on medical devices is low. In comparison, the pharmaceutical industry invests vast resources into development of new drugs. Most institutions lack experience and infrastructure on clinical studies involving a medical device. The different phases in development of a drug (phase I, II or III) may be applied for medical devices. Preclinical animal studies may also be relevant. Similar to studies on drugs, clinical studies to a different degree include aspects of safety (side effects), feasibility, efficacy and economic considerations. Depending on phase of study, different aspects are emphasized. The gold standard in drug studies is randomized controlled trials with placebo and double blinding. This design may not be possible to use at all in a study involving a medical device, unless there is some active component. Recommended standards or templates should be developed.

## 10. Conclusions

Measurement of physiological pressures is an important task in clinical practice. MEMS technology can provide new possibilities for monitoring of physiological parameters *inside* the human body. Such measurements are expected to improve the quality of medical diagnosis and treatment.

With continued miniaturization, application of implantable devices will imply less tissue damage. In clinical practice, this will be beneficial: Less need for anesthetics, reduced risk for bleeding and inflammatory reactions. High precision and frequency measurements may lead to a better understanding of physiological processes and risk factors for disease. Future development may combine measurements with devices for drug delivery, electric stimulation or alarm functions. Even with existing technology, wireless transfer of results from measurement may be a rapid and cost-effective method for follow-up of various diseases.

To succeed with the development of medical pressure sensors of clinical relevance, collaboration between technological experts and medical clinicians is of vital importance. Terminology should be standardized, and we suggest subcutaneously implantable for medical devices being totally inserted into the human body versus percutaneously implantable for devices with wires penetrating the skin for connection to an external energy source or readout unit. Improved guidelines for conducting clinical studies should be developed to incorporate specific aspects of implantable medical devices. There are several technical challenges to be solved, such as developing thin protective coatings offering sufficient protection against biological attack. Such coatings might support sensor stability and at the same time maintain the small-size advantage of implantable MEMS. Extensive cross-disciplinary research among MEMS developers, experts in material science and biologists are required to develop new biomaterials suitable as coating for implantable sensors for long-term use.

## Figures and Tables

**Table 1.** Site of operation and body reaction.

**Site of Operation**	**Example on Medical Application**	**Body Environment**	**Biological Effect**
Brain	Hydrocephalus monitoring	Cerebrospinal fluid with content of electrolytesSome sticky proteins	CorrosionProtein depositIn-growth in, e.g., choroid plexus
Eye	Glaucoma monitoring	Intraocular fluid with content of electrolytes	CorrosionPossible protein deposit
Intestinal tract		1.0 < pH < 9.0Aggressive enzymesIons	High corrosionEnzymatic reactions
Urinary tract/bladder	Cystometry	4.0 < pH < 9.0Content of electrolytesNormal conditions: no blood cellsNormal conditions: no sticky proteins	Corrosion
Intravascular	HypertensionHeart failure	7.0 < pH < 7.8Blood platelets, blood cells, and proteinsContent of electrolytes	CorrosionFibrous encapsulation
Subcutaneous, intramuscular (*i.e.*, connective tissue, cartilage, bone)	Compartment syndrome	4.0 < pH < 9.0Aggressive white blood cells (macrophages)Content of electrolytesOnly minor amounts of blood platelets and coagulation proteins	Corrosionmacrophages—foreign body attackSoft layer of white blood cellsFibrous encapsulationIn-growth in tissue with fibre, blood vessels and cells
Big joints	Research—prosthetic replacement and loosening	Synovial fluid with sticky proteinsContent of electrolytes	Protein layersCorrosion
